# The Roles of H3K9me3 Writers, Readers, and Erasers in Cancer Immunotherapy

**DOI:** 10.3390/ijms252111466

**Published:** 2024-10-25

**Authors:** Urszula Oleksiewicz, Monika Kuciak, Anna Jaworska, Dominika Adamczak, Anna Bisok, Julia Mierzejewska, Justyna Sadowska, Patrycja Czerwinska, Andrzej A. Mackiewicz

**Affiliations:** 1 Department of Cancer Immunology, Chair of Medical Biotechnology, Poznan University of Medical Sciences, 60-806 Poznan, Poland; 2 Department of Diagnostics and Cancer Immunology, Greater Poland Cancer Center, 61-866 Poznan, Poland; 3 Doctoral School, Poznan University of Medical Sciences, 60-812 Poznan, Poland; 4 Faculty of Physics, Adam Mickiewicz University, 61-614 Poznan, Poland; 5 Department of Health Sciences, The Jacob of Paradies University, 66-400 Gorzow Wielkopolski, Poland

**Keywords:** immunotherapy, cancer, H3K9me3, SETDB1, SUV39H1, LSD1, KDM3/4, HP1, KAP1

## Abstract

The interplay between cancer and the immune system has captivated researchers for a long time. Recent developments in cancer immunotherapy have substantiated this interest with a significant benefit to cancer patients. Tumor and immune cells are regulated via a wide range of molecular mechanisms involving intricate transcriptional and epigenetic networks. Epigenetic processes influence chromatin structure and accessibility, thus governing gene expression, replication, and DNA damage repair. However, aberrations within epigenetic signatures are frequently observed in cancer. One of the key epigenetic marks is the trimethylation of histone 3 at lysine 9 (H3K9me3), confined mainly within constitutive heterochromatin to suppress DNA accessibility. It is deposited at repetitive elements, centromeric and telomeric loci, as well as at the promoters of various genes. Dysregulated H3K9me3 deposition disrupts multiple pathways, including immune signaling. Consequently, altered H3K9me3 dynamics may modify the efficacy of immunotherapy. Indeed, growing evidence highlights the pivotal roles of various proteins mediating H3K9me3 deposition (SETDB1/2, SUV39H1/2), erasure (KDM3, KDM4 families, KDM7B, LSD1) and interpretation (HP1 proteins, KAP1, CHD4, CDYL, UHRF1) in modulating immunotherapy effectiveness. Here, we review the existing literature to synthesize the available information on the influence of these H3K9me3 writers, erasers, and readers on the response to immunotherapy.

## 1. Introduction

The relationship between cancer and immunity has been of interest to researchers for many years. The immune system has a pivotal role in defending the body from pathogens, non-self, or malignant cells. The immune system recognizes tumor-specific antigens (TSAs) and tumor-associated antigens (TAAs) and therefore defeats most nascent cancers. However, some malignant cells are able to evade elimination by the immune system [1]. There are several mechanisms employed by cancer cells to evade the antitumor immune response. Some aim to make cancer cells invisible to the immune system (e.g., via loss of antigenicity), while others induce resistance to killing by activated immune cells [2]. The development of immunotherapies that overcome cancer cell resistance to the immune system or boost patient immunity against cancer is of paramount importance in current research in oncology [3].

The phenotype of cancer or immune cells is driven by a plethora of pathways that are dependent on gene expression networks active at specific cellular states. These networks, in turn, are regulated and stabilized by various epigenetic mechanisms, which frequently are aberrant in cancer cells. Exposure to external and internal stressful factors, such as oncogenic signaling, hypoxia, nutrient deprivation, radiation, or chemotherapy, is associated with dynamic changes in epigenetic programs in all cells affected. These changes influence not only cancer cells but also a variety of cells within the tumor microenvironment (TME), including immune cells that orchestrate complex, context-driven defense reactions [4]. While epigenetic alterations are reversible, they may be utilized by cancer cells to adapt to alterations within their TME [5]. Epigenetic modifications may also drive immune cell identity, supporting their differentiation [6,7,8]. Mammalian epigenetic mechanisms include a wide portfolio of elements that modulate chromatin accessibility, allowing for the activation or repression of gene expression. One of the well-established markers of repressive epigenetic state blocking gene transcription is the trimethylation of lysine 9 at histone 3 (so-called H3K9me3). It occurs within heterochromatic regions, inhibiting the expression of various genes and repressing endogenous retroviruses and other repeat elements. Misbalanced H3K9me3 deposition affects multiple pathways, including immune-related signaling [9,10,11,12] and, as such, it may affect the outcome of immunotherapy. Indeed, growing evidence suggests that proteins involved in H3K9me3 deposition (i.e., writers), removal (i.e., erasers), and interpretation (i.e., readers) may be involved in the response to immunotherapy. In the sections below, we will summarize the available literature demonstrating how the writers, erasers, and readers of H3K9me3 may modulate the effects of immunotherapy.

## 2. The Interplay Between Cancer and the Immune System: Mobilizing Immune Evasion Mechanisms

While cancerous cells may appear in the body due to sporadic mutations or epimutations, an appropriately working immune system may recognize and eradicate them. Such a phenomenon is called immunosurveillance. The immune system recognizes TSAs and TAAs and defeats most nascent cancers. However, during cancer evolution, some cells may acquire avoidance mechanisms to escape the immune system.

One such mechanism can be described as cancer immunoediting. In the first elimination phase, immune effector cells eliminate cancer cells. Later, in the equilibrium stage, tumor cells escape being killed by immune cells, but the overall growth of the tumor is restricted by adaptive immunity. In the final stage of escape, the immune system can no longer eradicate tumor cells, leading to tumor progression [1]. Some escape mechanisms render cancer cells indistinguishable to immune cells, e.g., due to epigenetic repression of TAA or TSA expression, resulting in the loss of antigenicity [2]. Also, the loss of major histocompatibility complex I (MHC I) antigen presentation machinery (APM) results in defects of antigen presentation to T lymphocytes [2].

Furthermore, malignant cells may increase the expression of inhibitory ligands of immune checkpoint receptors on the surface of immune cells. Checkpoint blockade protects against autoimmunity and overactivation during infection [13]. To induce elimination, a T-cell receptor (TCR) binds an antigen presented by MHC molecules. However, any interaction between inhibitory receptors and their ligands blocks T cells from killing the attached cell. The two most significant immune checkpoint axes are programmed cell death-1 (PD-1) and cytotoxic T lymphocyte-associated protein 4 (CTLA4). Cancer cells are able to evade elimination by expressing the PD-1 ligand (PD-L1) that binds the PD-1 receptor on T cells to block its cytotoxicity [14]. While the PD-1 axis regulates T cells in peripheral tissues, the CTLA4 axis coordinates the activation of naive T cells in the lymph nodes. Antigen-presenting cells (APCs) express B7-1 (CD80) and B7-2 (CD86) that bind to CD28 on T cells, thus giving co-stimulatory signals that are crucial for activation. CTLA4 competes with CD28 to inhibit stimulation. The overexpression of CTLA4 on T cells may be induced by chronic inflammation and other TME features [15].

TME is a sophisticated ecosystem composed of tumor cells, stroma (with pro- and anti-cancer immune cells), and extracellular molecules. Altered vasculature depresses immune cell infiltration, whereas hypoxia within the tumor mass suppresses immune cell functions. Cancer cells can attract immunosuppressive cells to weaken the immune response [16]. Myeloid-derived suppressor cells (MDSCs) have an immunosuppressive effect on T cells and natural killer (NK) cells. They enhance angiogenesis and invasion by producing, e.g., interleukin 10 (IL-10), transforming growth factor β (TGFβ), or reactive oxygen species [17]. Tumor-associated macrophages (TAMs) also secrete proangiogenic factors and immunosuppressive cytokines (TGFβ, IL-6, IL-10, tumor necrosis factor α [TNFα]). TAMs are able to activate immune checkpoint blockade by expression of PD-L1 or B7 homologs to suppress cytotoxic CD8+ T lymphocytes (CTLs) and, concomitantly, recruit regulatory T lymphocytes (Tregs) [18]. Tregs exert various mechanisms of immunosuppression, including competitive IL-2 consumption, CTLA4 activation, cytokine production (IL-10, IL-35, TGFβ), granzyme B, and perforin secretion to destroy effector cells [19]. Tumor-associated neutrophils (TANs) modulate PD-1/PD-L1 signaling and release arginase 1 to inhibit T-cell proliferation [20]. Furthermore, it should be noted that on the molecular level, signaling pathways associated with immune evasion involve NF-κB, the MAPK cascade, and pathways dependent on PI3K/AKT, JAK/STAT, Wnt/β-Catenin, TGFβ, Notch, and Hippo [21].

## 3. Immunotherapy: From Concept to Clinical Usage

**Figure 1:**
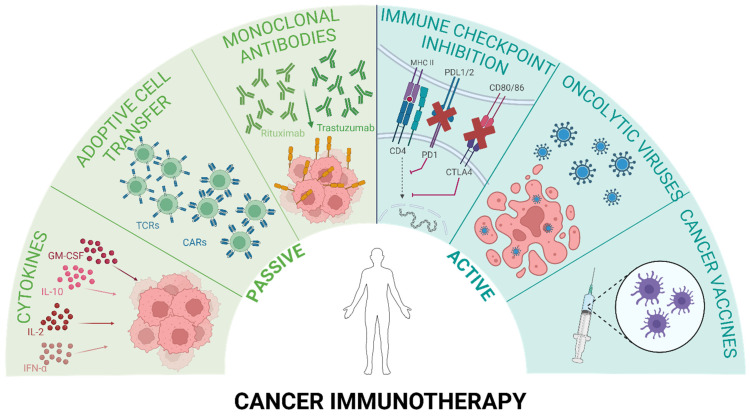
Passive and active forms of cancer immunotherapy. Passive methods include cytokine administration (e.g., IFNα, IL-2, IL-10, GM-CSF), adoptive cell transfer with T cells that are genetically engineered with specific T-cell receptors (TCRs) or chimeric antigen receptors (CARs), and monoclonal antibody targeting TSAs or TAAs. Active immunotherapy involves immune checkpoint blockade (ICB), oncolytic viruses, or cancer vaccines based on DNA, RNA, peptides, or whole cells. Created in BioRender. Oleksiewicz, U. (2024) BioRender.com/r45v820, accessed on 13 September 2024.

Stimulation of a patient’s immune system and restoration of its natural mechanisms to eradicate cancer cells are the fundamental principles of immunotherapy. Currently, evolving immunotherapeutic strategies can be grouped into two categories, as depicted in Figure 1. One approach is passive immunotherapy, where molecules are administered to a patient whose immune system cannot produce them autonomously or their secretion is insufficient. The second approach, active immunotherapy, involves direct stimulation of the immune response toward tumor cells. Both approaches can be specific or non-specific [3].

The concept of passive (or adoptive) immunotherapy encompasses agents that exhibit antitumoral activity. Unlike active immunotherapy, passive immunotherapy does not stimulate the patient’s immune system. Instead, it directly or indirectly targets cancer cells through the administration of “ready-to-use” immune system components, such as cytokines, antibodies, or immune cells [3]. One of the initially invented anti-cancer immunotherapies was adoptive cell therapy (ACT), which involves the infusion of activated immune cells into the patient’s bloodstream. There are two main groups of immune cells employed in ACT: adoptive tumor-infiltrating lymphocytes (TILs) and genetically engineered T cells with specific T-cell receptors (TCRs) or chimeric antigen receptors (CARs). Incorporating these engineered receptors improves cancer cell recognition and eradication [3]. Currently, there are six CAR T-cell therapies approved by the Food and Drug Administration (FDA): B-cell maturation antigen (Abecma, Carvykti) for the treatment of multiple myeloma, and CD-19 (Breyanzi, Kymriah, Tecartus, Yescarta) for the treatment of B-cell malignancies [22].

Cytokines were the first molecules to be used in immunotherapy. These signaling proteins secreted mostly by immune cells influence the growth and differentiation of blood cells and orchestrate the immune response. Several cytokines can hinder cancer growth by stimulating the cytotoxic activity of immune cells against tumor cells or by directly inhibiting proliferation and promoting apoptosis in cancer tissue [23]. The first cytokine approved by the FDA, interferon α (IFNα)—Intron A, induces antitumor immunity by stimulating T cells, NK cells, and dendritic cells (DCs). It also causes pro-apoptotic, anti-proliferative, and anti-angiogenic effects to treat multiple cancer types [24]. The second cytokine drug—IL-2 (Proleukin)—activates NK and T cells simultaneously, promoting T cells to expand and produce interferon γ (IFNγ) [25]. Both IFNα and IL-2 are exploited in the treatment of leukemia, renal cell carcinoma (RCC), and melanoma. Other cytokines, such as IL-10, IL-12, IL-15, IL-21, and granulocyte-macrophage colony-stimulating factor (GM-CSF), are undergoing clinical trials. Still, none of them have been approved yet due to several immune-related adverse effects [23].

The largest class of cancer immunotherapies used in clinics is antibody therapy. Antibodies bind specific antigens present on the cell surface and recruit effector immune cells (e.g., macrophages, NK cells) to initiate antibody-dependent cell-mediated phagocytosis (ADCP) or antibody-dependent cell-mediated cytotoxicity (ADCC). Antibodies can also activate the classical component cascade or act as drug delivery agents by carrying conjugated chemotherapeutics for targeted therapy. Immunotherapeutic antibodies used in clinics target cell antigens, such as CD20 in chronic lymphocytic leukemia (Obinutuzumab, Ofatumumab); CD20 in B-cell lymphoma (Rituximab); cell receptors, e.g., human epidermal growth factor receptor 2 (HER2) in breast cancer (Trastuzumab, Pertuzumab); as well as epidermal growth factor receptor (EGFR) in colorectal cancer and head and neck squamous cell carcinoma (HNSCC) (Cetuximab) [26].

Monoclonal antibodies can also be employed in active immunotherapy for checkpoint inhibitory therapy. Along with cancer vaccines and oncolytic viruses, they aim to restore the patient’s immune responses toward the tumor [3]. The FDA has approved monoclonal antibodies for the two abovementioned checkpoints (CTLA4, and PD-1 axes). Ipilimumab and Tremelimumab bind CTLA4 molecules to restrain them from binding to CD80 and CD86 antigens. Blocking the PD-1 axis can be achieved with anti-PD-1 antibodies (e.g., Nivolumab, Retifanlimab, Toripalimab, Pembrolizumab) or anti-PD-L1 antibodies (Atezolizumab, Avelumab, Durvalumab). These drugs have proved to be effective in eradicating several solid tumors, including metastatic melanoma, lung cancer, and kidney cancer [27].

Another promising approach is to potentiate the antitumor response with cancer vaccines. Currently studied cancer vaccines can be based on peptides/proteins, nucleic acids (DNA or RNA), or whole cells that aim to stimulate the immune response toward specific antigens present in cancer cells [28]. The FDA has approved two vaccines for cancer treatment: Bacillus Calmette–Guérin (BCG) and Sipuleucel-T. BCG is a live attenuated vaccine form of *Mycobacterium bovis* that is used to induce a tumor-specific immune response in non-muscle-invasive bladder cancer [29]. Sipuleucel-T, utilized in prostate cancer therapy, consists of the patient’s dendritic cells expanded ex vivo and activated with a known TAA: prostatic acid phosphatase (PAP) [30].

Oncolytic viruses are an emerging class of cancer immunotherapeutics. They preferably infect and destroy tumor cells rather than healthy cells. The anti-cancer effect is enhanced by newly released viral particles, whereas ongoing oncolysis boosts the immune response in the TME. Imlygic-talimogene laherparepvec (T-VEC) is the only FDA-approved viral drug to date that is effective in recurrent melanoma. It comprises Herpes Simplex Virus 1 (HSV-1) genetically modified to express GM-CSF [31].

Although immunotherapeutic approaches are very promising, they are still prone to failure. Thus, it is crucial to search for additional modulators that may support the efficacy of current immunotherapies. Improved understanding of tumor biology and, more specifically, the pathways responsible for altered cancer immunogenicity, will help to generate novel treatment regimens. Growing evidence indicates that epigenomic changes are vital in regulating immune-related pathways. Notably, H3K9me3 is one of the key components in intricate immune signaling that has already been shown to be involved in the modulation of various immunotherapies. For example, H3K9me3-mediated repression of self-renewal and survival genes impairs T-cell activity and thus the efficacy of CAR T-cell-based therapies. This phenomenon can be overcome by the inhibition of H3K9 methyltransferases, such as SUV39H1, which was demonstrated to stimulate a stem-like phenotype and prolonged activity of CAR T-cells [32]. Moreover, targeting H3K9me3 modulators (e.g., KDM4A or SETDB1) may activate anti-tumor immunity and enhance the efficacy of PD-1 blockade immunotherapy [33,34]. Modulation of H3K9me3 brings hope and promise to the field of cancer therapy. Its potential and limitations are further discussed in this review.

## 4. Molecular Functions of H3K9me3 Epigenetic Mark

Changes in the epigenetic signature are essential components of signaling within the cell nucleus. They drive alterations within the chromatin structure, rendering it more open or tightened, thus supporting activation or suppression (respectively) of certain genomic locations. Altogether, these alterations affect the expression status of the given loci. However, they may also influence other activities of DNA, including DNA repair, recombinant homology, and replication. The best-characterized epigenetic modifications include DNA methylation and histone post-translational modifications that function in an orchestrated way to establish a specific epigenetic profile [5].

H3K9me3 is a well-characterized, conserved modification associated with a compact chromatin structure. It occurs predominantly within the constitutive heterochromatin compartment. This means that its signature is similar in most tissue types, in contrast to H3K27me3-rich facultative heterochromatin with a tissue-specific pattern. Growing evidence suggests that the H3K9me3 profile is largely disturbed in cancer cells [5]. In normal cells, H3K9me3 marks various genomic regions, including repeated sequences (such as retroelements, telomeres, and centromeres) and 3′ ends of homologous Krüppel-associated box zinc finger genes (KRAB-ZFPs) [35,36,37,38]. Furthermore, H3K9me3 may be found at the regulatory promoter regions of various genes, including immune-related genes such as IL-6, FosB proto-oncogene (FOSB), interferon regulatory factor 5 (IRF5), and others [12,39,40,41]. Moreover, it may be deposited within the promoters of lineage-unsuitable genes during embryogenesis [36,42]. Accordingly, its removal facilitates the reprogramming of differentiated cells toward stem or stem-like cells [43,44]. The latter phenomenon has also been described in the context of cancer cell reprogramming [45,46]. H3K9me3 modification is thus crucial for establishing cell fate, and a growing body of evidence supports its role in shaping the identity of immune cells and, consequently, the immune response.

### 4.1. Epigenetic Components of H3K9me3-Related Signaling: Writers, Erasers, and Readers

H3K9me3 deposition by its cognate methyltransferases leads to the recruitment of repressive protein complexes that foster the deletion of activating epigenetic marks and drive heterochromatization [47,48,49]. Moreover, the H3K9me3 mark will also guide the maintenance of heterochromatin within a given locus via dedicated hereditary mechanisms [50]. The appearance and removal of H3K9me3 are coordinated by highly specific enzymes. H3K3me3 is introduced by “writers”—histone methyltransferases—and deleted by “erasers”—histone demethylases. H3K9me3 function is mediated by its effector proteins, so-called “readers”, which may recruit other proteins to perform a specific action associated with H3K9me3, e.g., induction of heterochromatization, H3K9me3 maintenance [47,48,49,50].

In the human genome, there are several proteins with methyltransferase activity specific toward H3K9, including SETDB1/2 (SET domain bifurcated 1/2), Suv39H1/2 (suppressor of variegation 3-9 homolog 1/2), and G9A. Similarly to other histone methyltransferases (specific for other histone lysines), these proteins contain a catalytic SET (Su(var)3-9, enhancer-of-zeste, trithorax) domain responsible for transferring methyl groups from its donor, S-adenozylomethionine (SAM), to the amino group of lysine [51]. The proteins have high specificity toward the number of methyl groups to be transferred onto lysine. SETDB1/2 and SUV39H1/2 participate in di- and trimethylation, while G9A participates in the initial monomethylation [48]. As G9a does not act as a H3K9me3 methylotransferase, it will not be described in this review. H3K9me3 demethylases are less specific than methyltransferases. This group includes lysine-specific histone demethylase 1A (LSD1, flavin-dependent amine oxidase), lysine-specific demethylase (KDM3A-D and KDM4A-D), Jumonji C family members (utilizing 2-oxoglutarate as a substrate), and KDM7B (Fe^2+^-dependent hydroxylase utilizing 2-oxoglutarate and oxygen) [47,52]. These proteins may show specificity toward other methylated histone lysines (e.g., LSD1 demethylates H3K4me3) [47].

The H3K9me3 mark is recognized by various protein domains, such as chromodomain, plant homeodomain (PHD), tudor, ankyrin repeats, and WD40. These domains may be found in the proteins with different function, e.g., KRAB-associated protein 1 (KAP1), heterochromatin protein 1 (HP1), chromodomain-helicase-DNA-binding protein 3/4 (CHD3/4), chromodomain Y-like protein (CDYL), and ubiquitin-like, containing PHD and RING finger domains 1 (UHRF1) [49]. KAP1 and HP1 are co-factors in a repressive complex tethered to specific DNA sequences by KRAB-ZFP factors via their zinc finger domain. KAP1 protein functions as a scaffold that recruits histone deacetylase (HDAC) from the nucleosome remodeling and deacetylation (NuRD) complex, SETDB1 methyltransferase, and HP1, which induces heterochromatization [53]. UHRF1 may interact with H3K9me3 and hemimethylated DNA, promoting methylation on a newly synthesized strand [49]. CHD3/4/5, the components of the NuRD complex, are ATP-dependent chromatin remodelers with helicase activity, which play a role in gene repression [54,55]. Other proteins may directly bind H3K9me3, as demonstrated with the three-yeast hybrid assay. However, in the case of some of these proteins, the functional significance of such interaction awaits further clarification [56].

### 4.2. H3K9me3 Control over Immune-Related Genes

**Figure 2:**
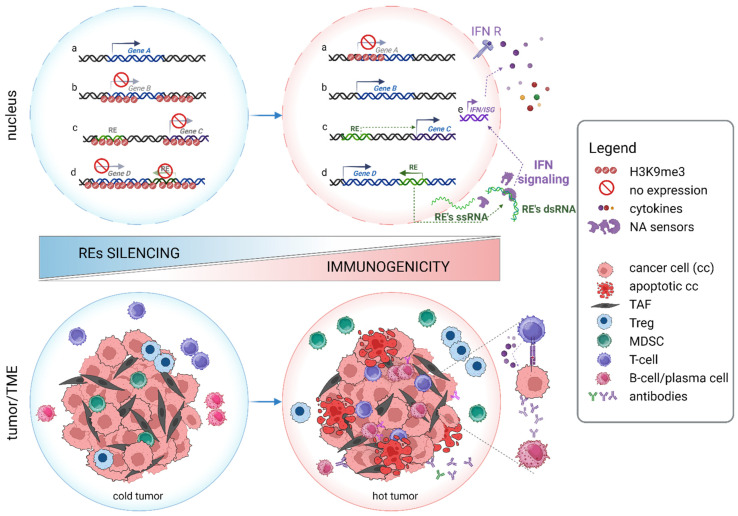
H3K9me3 regulation of immune genes and its effect on tumor immunogenicity. Internal or external stimuli induce changes within the H3K9me3 landscape in affected cells. **Upper panel**: Direct deposition or removal of H3K9me3 results in the silencing or expression of immune-related genes (Genes A and B, respectively; a and b). Loss of H3K9me3 also activates repetitive elements (RE: ERVs, LINEs, and SINEs; shown in green). As sources of promoters and regulatory sequences, they affect the expression of nearby immune genes (Gene C, trans-effect; c). When inserted within the activated gene (Gene D), they alter the transcript by introducing alternative splicing sites, polyadenylation signals, stop codons, etc. (cis-effect; d). Transcribed REs are recognized by host nucleic acid sensors, leading to interferon signaling activation d, e. Affected cells produce interferon (IFN), which further acts through autocrine and paracrine pathways to modulate the immune response. Consequently, different interferon-stimulated genes (ISGs, e), including cytokines, are expressed in various cell types. **Lower panel:** Loss of H3K9me3 within REs transforms non-immunogenic (cold) tumors into immune-responsive (hot) tumors. Cold tumors are characterized by the presence of immune-suppressive cells, i.e., myeloid-derived suppressor cells (MDSCs) and regulatory T cells (Tregs). Hot tumors show an inflammatory phenotype with the accumulation of proinflammatory cytokines, high levels of infiltrating T lymphocytes and B cells, and enhanced production of tumor-reactive antibodies. RE: repetitive element, ss/ds: single/double strand, IFN: interferon, IFN R: interferon receptor, NA: nucleic acid, TME: tumor microenvironment, and TAF: tumor-associated fibroblast, cc: cancer cell. Gene examples: Genes A: *PD-1*, *CTL4*, *LAG-3*; Gene B: *FAS*, 1; Genes C: *CSF1R*, *IRF5*; Genes D: *MET*, *ALK*, *ERBB4*, *SLCO1B3*. See text and Table 1 for more details. Created in BioRender. Oleksiewicz, U. (2024) BioRender.com/g80u272, accessed on 10 October 2024.

The presence of H3K9me3 serves as a distinguishing feature of heterochromatin, signifying regions of the genome that are transcriptionally inactive. Apart from repetitive elements, centromeric and telomeric sequences, H3K9me3 may also mark the genes that are silenced in a highly context-dependent manner. For instance, H3K9me3 modification plays a crucial role in guiding the lineage commitment of T helper 2 lymphocytes (Th2) by suppressing Th1-specific genes, and it governs adequate, antigen-specific CD8+ T-cell response [6,7,8]. It also contributes to Th17 differentiation, as well as B cell development and commitment [57,58]. Regulation of immune genes is thus multidirectional and dynamic, encompassing both short- and long-term changes that bridge periods of cellular stress. It might also be a direct process in which H3K9me3 is imposed straightforwardly on target genes, mostly within their promotor regions. Alternatively, it might involve epigenetic remodeling of different repetitive sequences, the activation of which upregulates various signaling pathways linked to immune processes [36] (Figure 2).

### 4.3. Direct Regulation of Immune-Related Genes by H3K9me3

An example of a direct mode of H3K9me3 action is the silencing of Fas (Fas cell surface death receptor) in metastatic colorectal cancer (CRC). Fas protein binds Fas ligand (FasL) expressed by T cells and NK cells, including activated cytotoxic T lymphocytes (CTLs). Fas–FasL binding initiates apoptosis of cancer cells; hence, progressive downregulation of Fas in cancer enables immune evasion [10]. Moreover, in CRC, H3K9me3 negatively regulates FOSB transcription factor, leading to PD-L1 upregulation (via miR-22/BATF3 axis) and, consequently, a reduction in T-cell cytotoxicity [12]. Similarly, direct H3K9me3-mediated immune escape was reported in breast and pancreatic cancer. Notably, in each case, it engaged different molecular mechanisms. In breast cancer, a decrement of H3K9me3 modification was reported on promoters of key immune checkpoints, namely *PD-1*, *CTLA-4*, and *lymphocyte activating 3* (*LAG-3*) [9]. By contrast, in pancreatic cancer, increased H3K9me3 levels within the *BCL2 antagonist/killer 1* (*BAK1*), *BCL2 associated X (BAX)*, and *BLC2-like 11* (*BCL2L11)* gene promoters were shown to inhibit the expression of these apoptotic effectors [59]. Downregulation of BAX not only facilitated cancer cell proliferation but also affected the tumor immune microenvironment by decreasing the infiltration level of CD8+ T cells [60].

**Table 1:** Alterations of H3K9me3 levels at chosen targets with the potential to affect tumor immunogenicity. Only the most relevant pathways and processes for tumor immunity are listed. Legend: ↓ decrease in H3K9me3 level; ↑ increase in H3K9me3 level; ↓↑ decrease or decrease in H3K9me3 levels, which varies depending on cancer type and multiple conditions, e.g., oxygen conditions (see references for more details).

H3K9me3 Level	H3K9me3 Targets	Pathway/Process Affected	References
↓	*Retroelements (REs)*	Viral mimicry response, onco-exaptation of RE-derived elements, tumor antigen presentation	[61,62,63,64,65]
↓	*PD-1, CTLA-4 and LAG-3*	Immune checkpoint signaling	[9]
↑	*BAK1, BAX, BCL2L11*	Apoptosis, CD8+ T-cell infiltration	[59,60]
	*INK4B, WAF1*	TGFβ signaling, cell cycle	[66,67]
	*FAS*	Fas–FasL signaling pathway	[10]
	*FOSB*	BATF3/PD-L1 signaling	[12]
↓↑	*MEIS1, HOXA*	Metabolic pathway, ERK1/2, Notch, Smad pathways, apoptosis	[68,69,70]
↓↑	*HIF1*	EMT, autophagy, metabolic reprogramming	[71]

### 4.4. Indirect Regulation of Immune-Related Genes by H3K9me3

Immune genes are also indirectly regulated through epigenetic make-up of repetitive sequences, such as retroelements (REs) (Figure 2; c, d). These include long terminal repeats (LTRs), represented by endogenous retroviruses (ERVs), and non-LTRs with long interspersed elements (LINEs) and short interspersed elements (SINEs) [72]. While once viewed as genetic relics or “junk DNA”, research spanning the past two decades has highlighted their immense roles in genome evolution and regulation of gene expression. Indeed, as a source of *cis*-regulatory sequences (promoters and enhancers), alternative cryptic splicing sites, and polyadenylation signals, REs have the potential to influence the expression of proximal and distant genes. Some of the examples of the genes regulated via RE-dependent mechanisms include *colony-stimulating factor 1 receptor (CSF1R); interferon regulatory factor 5 (IRF5)*; *MET proto-oncogene, receptor tyrosine kinase (MET); anaplastic lymphoma kinase (ALK); Erb-B2 receptor tyrosine kinase 4 (ERBB4); and solute carrier organic anion transporter family member 1B3 (SLCO1B3)* [38,73] (Figure 2; c, d). When activated, they can “jump into” new loci, posing a risk of DNA mutation and genome instability [74,75]. Therefore, REs are tightly controlled and normally silenced in healthy somatic cells at DNA and histone levels [57,76]. However, their expression is often observed in cancer, mostly triggered by the loss of repressive marks [76,77,78].

H3K9me3 is a dominant epigenetic repressor of REs in the human genome. Loss-of-function studies have shown that different heterochromatin-associated proteins, such as, e.g., SETDB1, SUV39H1, KAP1, the human silencing hub (HUSH) complex, enhancer of rudimentary homolog (ERH) or KDM5B, engage H3K9me3 to repress distinct types of repetitive elements via alternate complexes and in a context-dependent manner [79,80,81].

Reactivation of REs often leads to the cascade of molecular events mirroring the antiviral immune response driven by interferons (Figure 2) [76,77,78]. First, retrotransposon transcripts are recognized by pattern recognition receptors (RNA sensor [RIG-I], interferon-induced helicase C domain-containing protein 1 [MDA5], and cyclic GMP-AMP synthase [cGAS]) functioning as foreign DNA sensors. This subsequently leads to the activation of mitochondrial antiviral-signaling protein (MAVS) and/or stimulator of interferon genes (STING) and, finally, to the expression of interferon stimulatory genes (ISGs) via the IRF3/7-dependent mechanism [82,83,84]. This phenomenon, known as viral mimicry, ranks the epigenetic modifiers of REs to a top position on the list of promising targets in cancer therapy. Indeed, increasing data demonstrate that aberrant expression of REs renders tumors more immunogenic and more susceptible to immunotherapy (Figure 2) [63,65].

An interesting study by Cuellar et al. demonstrated that although the loss of SETDB1 in acute myeloma (AML) cells resulted only in a modest global decrement in H3K9me3 levels; it was associated with immediate de-repression of ERVs, LINEs, and satellite repeats, followed by activation of the type I IFN antiviral response and apoptosis [62]. Further research revealed that AML patients can be stratified based on the RE to gene expression ratio and that RE activation positively correlates with better prognosis [85]. Another study showed that KDM7B stabilizes SETDB1, thus enhancing H3K9me3 levels at REs. Low RE activity, in turn, is associated with diminished tumor sensitivity to immune checkpoint blockade (ICB) [86]. Consistently, different studies on lung, skin, bladder, ovary, colon, and other tumor types show that blocking of SETDB1 and other H3K9me3-related proteins sensitizes tumors to ICB treatment [33,34,79,81,87,88,89]. For example, in murine melanoma and lung cancer models, Setdb1 knockout evoked the activation of REs and neighboring genes, leading to augmented expression of retroelement-encoded MHC I peptides targeted by T-cell cytotoxicity [79]. Inhibition of SUV39H1 in different types of cancer also stimulates interferon signaling, enhances cancer cell immunogenicity, reduces the expression of inhibitory receptors, and generally improves the efficacy of T-cell therapy [11,32,90]. These and other reports on the involvement of H3K9me3 modifiers and readers will be described in more detail in the upcoming sections.

## 5. H3K9me3 Methyltransferases and Their Involvement in Immunotherapy

### 5.1. SETDB1/2

SETDB1 and SETDB2 (SET domain bifurcated histone lysine methyltransferase 1 and 2), next to SUV39H1 and SUV39H2, belong to the SET-domain methyltransferases family. The characteristic function of SETDB1 is the deposition of H3K9me2 and H3K9me3 at endogenous and exogenous retroelements to mediate their silencing, thus preventing retrotransposition and preserving genome stability [91]. An increasing number of observations indicate SETDB1 involvement in tumor initiation and progression, the antitumor immune response, and gene expression control [92,93]. An analysis of TCGA (The Cancer Genome Atlas) datasets suggested that amplification of *SETDB1* supports tumors in immune escape [79]. Furthermore, a pan-cancer analysis indicated that SETDB1 is overexpressed in the majority of cancers, suggesting its oncogenic character. The study also pinpointed that the expression of SETDB1 correlates with immune and molecular cancer subtypes, as well as immune checkpoint- and HLA-related genes (human leukocyte antigen-related genes) [92].

**Figure 3:**
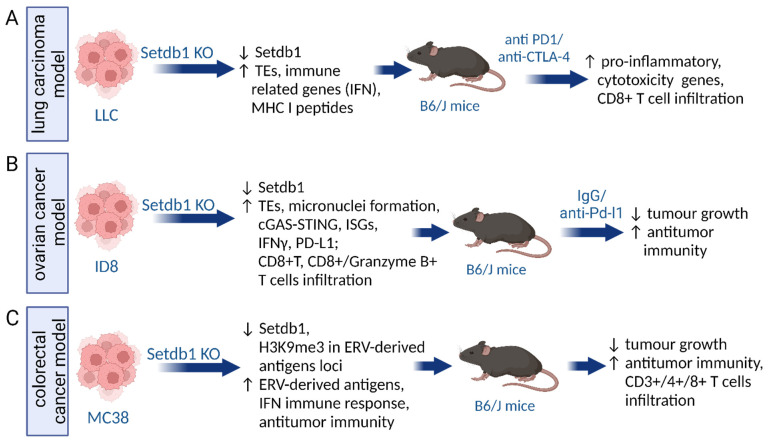
SETDB1 loss augments immunotherapy efficiency in murine models of lung carcinoma (**A**), ovarian cancer (**B**), and colorectal cancer (**C**). ↑ Upregulation/Increase, ↓Downregulation/Decrease, KO (knockout, LLC (Lewis lung carcinoma), ERV (endogenous retroviruses), TEs (transposable elements), MHC I (major histocompatibility complex class I), IFN (interferon), PD-L1 (programmed death ligand-1), ISGs (interferon-stimulated genes), cGAS–STING (cyclic GMP–AMP synthase-stimulator of interferon genes), and REs (retroelements). Created in BioRender. Oleksiewicz, U. (2024) BioRender.com/a02b218, accessed on 10 October 2024.

Interestingly, SETDB1 can be the target accompanying immune checkpoint blockade (ICB) treatments (Figure 3). Griffin and colleagues examined the influence of epigenetic modulators on ICB in mouse melanoma and lung carcinoma models (Figure 3A). Their observations revealed that the loss of Setdb1 elevated ICB treatment efficacy by sensitizing tumor cells. A comprehensive analysis of regions affected by Setdb1 knockout in these cells revealed 543 common loci showing the activation of retroelements and nearby genes. These domains were rich in segmental duplications and immune-related gene clusters, such as a species-specific cluster of IFN, the lymphocyte antigen 6 (Ly6) family, and non-canonical major histocompatibility complex class I (MHC I). One of the SETDB1-dependent loci contained genes activating NKG2-D type II integral membrane protein (NKG2D receptor) on immune cells (NK and CD8+ T cells). Ablation of SETDB1 in this study did not induce the antiviral IFN pathway but unleashed the expression of retroelement-encoded MHC I peptides, both in mouse and human cancer cells. Some of these peptides were further shown to be targets of T-cell immunity, as evidenced by increased T-cell infiltration and increased expression of pro-inflammatory and cytotoxicity genes [65].

The analysis of TCGA datasets of ovarian cancer indicated a negative correlation between SETDB1 or TRIM28 and CD274 expression (encoding PD-L1 immune checkpoint). The efficiency of ICB therapy increased after the loss of the SETDB1–TRIM28 complex in ovarian cancer cell lines due to cGAS–STING pathway activation. It resulted in PD-L1 overexpression, IFNγ release, and more effective infiltration of effector CD8^+^ T and CD8^+^/Granzyme B^+^ T cells. Thereby, tumor burden decreased after Setdb1 KO in immunocompetent mice (Figure 3B). Combining anti–PD-L1 treatment and Setdb1 KO gave synergistic results in elevating CD8^+^ T-cell infiltration [33]. Another study also observed the activation of antitumor immunity upon the loss of Setdb1 [94]. CRISPR/Cas9-mediated KO of Setdb1 or its interacting partner Atf7ip (activating transcription factor 7-interacting protein) in multiple murine tumor models increased antigen expression and resulted in retardation of tumor growth in immunocompetent mice (Figure 3C). Additionally, CD3^+^, CD4^+^, and CD8^+^ T-cell infiltration was elevated. Importantly, the observations highlighted the overexpression of ERV-derived antigens and activation of interferon pathways, probably through H3K9me3 deficiency within ERV and ERV-derived antigen regions. Inhibition of Setdb1 and Atf7ip yielded resembling consequences, resulting in restored immunosurveillance and increased antitumor immunity [94].

The contribution of SETDB1 to immune evasion was also evidenced in melanoma and colorectal mouse cell lines, however via interaction with KDM5B (lysine-specific demethylase 5B), a histone demethylase targeting H3K4me3 [81]. Although the study by Zhang and colleagues focused mainly on the effect of KDM5B knockout on the anti-cancer immune response, the authors showed that KDM5B recruits and stabilizes SETDB1 to silence its targets, including REs. Decreased expression of KDM5B resulted in reduced H3K9me3 deposition at REs and their subsequent de-repression. This, in turn, stimulated cytosolic RNA and DNA sensors and, consequently, the type I interferon response. Altogether, these molecular events mediated tumor growth inhibition and augmented the efficiency of anti-PD-1 therapy [81].

Our understanding of the involvement of SETDB2 in cancer immunity is still limited. Notably, SETDB2 belongs to interferon-stimulated genes (ISG). Schliehe et al. demonstrated SETDB2 activation upon type I interferon signaling in response to influenza virus infection, and STAT1 (signal transducer and activator of transcription 1) and IRF7 were involved in the process. SETDB2 repressed nuclear factor kappa-light-chain-enhancer of activated B cell (NF-κB) target genes with antibacterial activities, including neutrophil attractant C-X-C motif chemokine 1 (CXCL1), and impaired the inflammatory response. It was shown that lowered SETDB2 levels led to decreased H3K9me3 levels at the CXCL1 promoter [41]. In addition, IFN signaling and SETDB1/SETDB2 expression were enhanced upon exposure to chemotherapeutic agents or targeted therapies in melanoma, lung, and colon cancer cell lines, a phenomenon associated with therapy resistance. Inhibition of these enzymes reduced H3K9me3 levels and reversed multidrug tolerance [41,95].

### 5.2. SUV39H1/KMT1A

Suppressor of variegation 3-9 homolog 1 and 2 (SUV39H1 and SUV39H2) are other lysine methyltransferases (KMTs) specific for H3K9 di- and tri-methylation that lower chromatin availability and evoke gene silencing [48,96]. SUV39H1, also known as KMT1A, is engaged in the differentiation of CD4^+^ and CD8^+^ T-cell subtypes through H3K9me3-mediated gene silencing [7,8]. In carcinogenesis, SUV39H1 performs two opposite functions: it may act as a suppressor or oncogene. Overexpression of SUV39H1 is evidenced in bladder, liver, and colon cancer, melanoma, and clear cell renal carcinoma [96,97,98,99]. A growing number of reports strongly suggest that SUV39H1 may be an interesting target for anti-cancer therapy. For example, SUV39H1 silencing was shown to promote ferroptosis, as demonstrated in clear cell renal cell carcinoma [96]. Moreover, inhibition of SUV39H1 with chaetocin potentiated ionizing radiation (IR) therapy due to chromatin relaxation and de-repression of major histocompatibility complex class I-related chains A and B (MICA/B) upon IR-induced double-strand breaks. MICA/B are ligands for surface receptors on immune cells (e.g., NK and CD8^+^ T cells). Thus, MICA/B overexpression via chromatin relaxation (through HDAC/Suv39H/G9A axis) enhances the immune cell response, supporting radiotherapy [100].

Most colon cancers are insensitive to ICB immunotherapy with anti-PD-1 and anti-PD-L1 antibodies [101]. Lu and colleagues synthesized a new agent, F5446, a small molecule working as an SUV39H1 inhibitor. SUV39H1 was chosen as a therapeutic target since its expression is significantly higher in colon cancer cells and CTLs compared to normal cells. Moreover, H3K9me3 was found to be elevated within the promotors of SUV39H1 effector genes (granzyme B, perforin 1, FASLG, IFNγ) in CTLs [102]. These factors are crucial components of the immunosurveillance system, initiating apoptosis and inhibiting tumor growth [103]. SUV39H1 inhibition led to increased expression of granzyme B, perforin, FasL, and IFNγ [102], while a number of tumor-infiltrating CTLs remained unchanged in MC38 and CT26 colon tumor models. These findings indicate that the effectiveness of F5446 relies on CTL activation via decreasing H3K9me3 deposition at the promoters of effector genes. Interestingly, the combination of F5446 and anti-PD-1 treatment did not have additive or synergistic effects in mice [102].

In the study by Shen and colleagues [11], SUV39H1 was shown to be recruited to chromatin via its cofactor, FBXO44 (F-box only protein 44). SUV39H1 and FBXO44 are parts of an epigenetic repressive complex that mediates the H3K9 trimethylation responsible for RE silencing. This type of modification at REs induced by SUV39H1 and FBXO44 is characteristic for cancer cells only. FBXO44/SUV39H1 inhibition boosted the immune response due to the activation of cytotoxic CD8^+^ T and NK cells and increased IFN-β release. Mechanistically, lower FBO44/SUV39H1 expression resulted in cancer cell death due to the induction of viral mimicry pathways stemming from RE de-repression, the presence of cytosolic dsRNA and dsDNA, and replication stress evoked by double-strand DNA breaks. As such, inhibiting SUV39H1 by F5446 in mouse breast cancer model augmented the sensitivity to anti-PD-1 treatment, reduced tumor progression, and increased survival. Of note, in contrast to many known methods, inhibitors of SUV39H1 could be a selective treatment in which normal cells are protected [11].

SUV39H1 inhibition was also demonstrated to support immunotherapy based on CAR-T technology [32,90]. An altered H3K9 methylation profile via SUV39H1 inactivation resulted in the reprogramming of 41BB-based chimeric antigen receptor T (BBz-CAR-T) cells, which acquired more self-renewing, stemlike properties that facilitate prolonged activity and persistence. Thus, H3K9 demethylation of BBz-CAR T cells reduced the risk of lung cancer in a mice model [90]. Similar outcomes were observed in leukemia and prostate cancer models [32]. Improved therapeutic effect (higher CAR-T expansion and tumor rejection rate) coincided with increased global chromatin accessibility with a concomitant decrease in the accessibility of a subset of inhibitory receptors and effector transcription factors (including transcription factor 7 [TCF7] and lymphoid enhancer-binding factor 1 [LEF1]). Lowered chromatin accessibility was associated with reduced expression of these genes. Thus, epigenetic reprogramming via SUV39H1 disruption may enhance the overall efficiency of adoptive T-cell therapy [32].

### 5.3. SUV39H2/KMT1B

SUV39H2, also known as KMT1B, mediates not only di- and tri-methylation of H3K9, but it may also methylate lysines within other proteins, including K322 in LSD1 and K134 in H2AX [104]. The data indicate that knockdown of SUV39H2 increased sensitivity to chemotherapy with doxorubicin in HeLa cells and to radiation of RERF-LC-AI cells [105]. Methylation induced by SUV39H2 influences cell cycle regulation, differentiation [106], carcinogenesis, invasion, and metastasis [107,108]. For example, SUV39H2 silences SLIT1 (slit guidance ligand 1) in an H3K9me3-dependent manner, and this is associated with oncogenic features in CRC, including metastasis and poor prognosis [108]. Overexpression of SUV39H2 is a common phenomenon in multiple cancer types, including hematopathies (e.g., leukemias and lymphomas), breast cancer, digestive system cancers (e.g., colorectal carcinoma), lung cancer, urinary and reproductive system cancers (e.g., prostate cancer), and Bowen’s disease [108,109,110]. Moreover, high levels of SUV39H2 are connected to poor prognosis in basal-like or triple-negative breast cancer, colorectal carcinoma, and esophageal squamous cell carcinoma (ESCC) [108,111].

Interestingly, an SUV39H2 peptide was identified as a novel cancer-testis antigen with immunogenic function. Kochin and colleagues analyzed the ligandomes of selected human colon and lung cancer cell lines. Among 403 ligands of HLA-A24 (one of the MHC I types), the SUV39H2 peptide appeared to be a tumor-associated antigen in colon cancer. Notably, SUV39H2 expression is present only in cancer cells but not in normal cells (apart from testis). A synthetic RF8 peptide encoded within the SUV39H2 sequence triggered a specific CTL response in HLA-A24-positive colon and lung cancer cell lines. Hence, SUV39H2 immunogenic peptides may serve as promising future candidates for targeted immunotherapy in various cancers [112]. In another study utilizing a model of trained immunity in monocytes, SUV39H2 was shown to contribute to reduced cytokine production. In the experimental setup, adherent monocytes were stimulated with BCG in the presence or absence of all-trans retinoic acid (ATRA), one of the agents registered for the treatment of acute promyelocytic leukemia. ATRA increased the levels of SUV39H2, which in turn induced H3K9me3 deposition within the promotors of several cytokine genes, thus resulting in their repression. By contrast, SUV39H2 inhibition caused higher IL-6 production [113].

In summary, the published reports indicate that SUV39H1/2 are promising targets for cancer treatment. They may support chemo-, radio-, and immunotherapies. Mechanistically, their function is mainly associated with the H3K9 methyltransferase activity that affects REs and various immune-related genes. However, SUV39H2 also demonstrates another mode of action, as its peptide was categorized as a cancer-testis antigen, eliciting a specific immune response.

## 6. H3K9me3 Demethylases

### 6.1. KDM3/JMJD1 Family of Histone Demethylases

The KDM3/JMJD1 family of histone demethylases, including KDM3A to KDM3D, plays a crucial role in regulating gene expression by removing mono- and di-methylation from H3K9. It has gained attention due to its roles in embryonic development, reproduction, stemness, differentiation, metabolism, and oncogenesis [114,115,116]. Among all of the members of the KDM3 family, the oncogenic function of KDM3A is most frequently reported in the literature, as it mediates cancer cell proliferation, survival, migration, and tumor metastasis [116,117].

**Figure 4:**
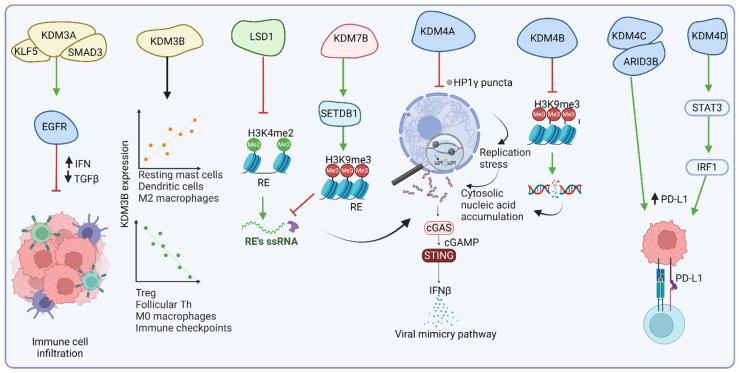
The influence of H3K9me3 histone demethylases on the interactions between cancer cells and the immune system. Most data indicate that H3K9me3 histone demethylases inhibit cancer immunogenicity. KDM3A hinders the immune response via upregulating EGFR expression with its co-activators: KLF5 and SMAD4. KDM4A and KDM4B protect against DNA replication stress, thus decreasing cGAS/STING signaling and the subsequent viral mimicry pathway. KDM4C and KDM4D are co-activators that support the upregulation of PD-L1 checkpoint molecules, thus mediating immune evasion. KDM7B and LSD1 guard low RE activity, thus turning off the viral mimicry response. KDM7B stabilizes SETDB1, which promotes higher H3K9me3 deposition at RE loci, whereas LSD1 removes the activating H3K4me2 mark from RE regions. Green arrows—activation, red—inhibition. Created in BioRender. Oleksiewicz, U. (2024) BioRender.com/s20b370, accessed on 19 October 2024.

Recent studies have shown that KDM3A functions as an epigenetic suppressor of the immunotherapy response in pancreatic ductal adenocarcinoma (PDA) [117]. ChIP-seq data revealed that KDM3A binding is enriched in the vicinity of Krüppel-like factor 5 (KLF5) consensus recognition motifs. Further analyses demonstrated that KDM3A operates together with KLF5 and SMAD family member 4 (SMAD4) to control the expression of a shared transcriptional network, including EGFR. The KDM3A/KLF5/SMAD4/EGFR axis (Figure 4, Table 2) coordinates the regulatory mechanism involved in maintaining a low population of T cells within the tumor microenvironment (TME), thus driving resistance to immunotherapies. Ablation of KDM3A, KLF5, SMAD4, or EGFR elevates the population of tumor-infiltrating T cells and dendritic cells and decreases myeloid cell infiltration. Such an alteration reprograms the TME to a T cell-inflamed state, enhancing sensitivity to combination immunotherapy [117].

KDM3A expression was also shown to play a role in colorectal adenocarcinoma (COAD). In the recent study reported by Kong and colleagues, KDM3A together with ENO3 served as an important biomarker for predicting metastasis. Using the KDM3A and ENO3 signature enabled efficient stratification of COAD patients into high- and low-risk groups. Importantly, the high-risk group presented increased expression of immune checkpoints and tumor mutation burden (TMB) [118]. Another study also demonstrated that high KDM3A expression is associated with poor prognosis in COAD patients [119]. This research revealed also that high risk tumors harbor a notable enrichment of APC co-inhibition, checkpoint pathways, HLA expression, para-inflammation, T-cell co-inhibition, and type I/II IFN responses. The high-risk tumors were also prone to augmented TMB, microsatellite instability, and overall TILs [119]. Moreover, an in-depth investigation into immune checkpoints unveiled significantly elevated expression of key checkpoint molecules (PD-L1, PD-1, and CTLA-4) in the high-risk cohort compared to the low-risk counterpart [118,119]. This heightened expression of immune checkpoints, in conjunction with substantial immune cell infiltration and the enrichment of immune-related pathways, underscored the potential sensitivity of high-risk groups to immunotherapeutic interventions.

Another member of the KDM3 family, KDM3B, was identified as one of the six independent prognostic genes in kidney renal clear cell carcinoma (KIRC). In contrast to KDM3A in PDA and COAD, KDM3B was downregulated in KIRC samples compared to normal tissues, which was also linked to poor outcomes in KIRC patients [115]. The study demonstrated that the low-risk group (with higher KDM3B expression) exhibited higher levels of resting mast cells, dendritic cells, and M2 macrophages while displaying lower levels of regulatory T cells, follicular helper T cells, and M0 macrophages as compared to the high-risk group (Figure 4, Table 2). Moreover, immune checkpoint molecules were downregulated in the low-risk group, indicating potential differences in the immune response between both groups [115]. Furthermore, a significant, positive correlation was detected between the expression of individual KDM3 family members and the infiltration levels of various immune cells, including B cells, CD8+ T cells, CD4+ T cells, macrophages, neutrophils, and dendritic cells, in hepatocellular carcinoma (HCC) [114]. Although KDM3C demonstrates anti-inflammatory properties by suppressing (NF-κB) signaling against oral bacteria [120], the molecular signaling by KDM3C in cancer settings remains largely unknown.

### 6.2. KDM4/JMJD2 Family of Histone Demethylases

The KDM4/JMJD2 family of histone demethylases includes KDM4A to KDM4D. These enzymes demethylate di- and tri-methylated lysines 9 and 36 on histone H3 (H3K9me2/3 and H3K36me2/3). They are implicated in cell cycle regulation, DNA damage response, and cancer progression (Figure 4). KDM4A, for instance, is frequently overexpressed in cancers, including head and neck squamous cell carcinoma (HNSCC). KDM4A ablation, especially when synergized with PD1 blockade therapy, was demonstrated to effectively inhibit SCC growth and metastasis and eradicate cancer stem cells. Mechanistically, the deletion of KDM4A evoked the formation of liquid-like condensates of HP1γ on heterochromatin enriched with H3K9me3 (Figure 4, Table 2). The presence of HP1γ puncta disrupted DNA replication, thus aggravating heterochromatin’s vulnerability to DNA replication stress in highly proliferative cancer cells. This process led to the accumulation of cytosolic DNA, followed by the activation of cGAS–STING signaling and the expression of interferon and Th1-related chemokines. Eventually, the signaling cascade mediated antitumor immunity by recruiting CD8+ T cells into the TME [34].

A recent study showed that targeting members of the KDM4 family (KDM4A-D) with the chemotherapeutic drug JIB-04 epigenetically triggered tumor-intrinsic innate immune responses and immunogenic cell death, resulting in remarkable treatment outcomes [121]. JIB-04 induces H3K9 hypermethylation, which impairs DNA repair and, similarly to the study described above [34], results in the accumulation of DNA damage, activation of the cGAS–STING pathway, IFN release, and, subsequently, restoration of immunosurveillance (Figure 4, Table 2). JIB-04 induces PD-L1 expression, which can be countered with PD-L1 blockade, providing a synergistic, antitumor effect. Interestingly, among all KDM4 family members, KDM4B ablation combined with PD-L1 blockade resulted in the most significant tumor inhibition. Moreover, unlike other chemotherapy agents, JIB-04 demonstrates tumor specificity and does not negatively impact the immune system. JIB-04 has shown effectiveness in preclinical models of various cancers, e.g., lung cancer, prostate cancer, and lymphoma. Further research and clinical trials are needed to fully explore its potential and determine its efficacy and safety in humans [121].

Another study revealed that KDM4C, recruited by ARID3B (AT-rich interaction domain 3B), drives PD-L1 expression in colorectal cancer stem cells, facilitating immune evasion. This activation is crucial for maintaining CRC growth and stem-like characteristics. Specifically, ARID3B recruits KDM4C to the promoter regions of ARID3B-regulated genes, such as Notch targets, to support H3K9me3 demethylation and activation of gene expression. Administration of a KDM4 inhibitor (NSC63819) augmented the global H3K9me3 pool and blocked the expression of ARID3B target genes, including Notch targets, PD-L1, and intestinal epithelial stem cell genes (Figure 4, Table 2). [122].

The last member of the KDM4 family, KDM4D, is also involved in regulating various pathways implicated in immune signaling. In colorectal cancer and hepatocellular carcinoma, KDM4D acts as a coactivator of Wnt/β-catenin, Hedgehog, HIF1, JAK-STAT3, and Notch pathways [123,124]. More specifically, in HCC with gain-of-function mutations in the *CTNNB1* gene (encoding β-catenin), the KDM4D complex with β-catenin triggers the expression of MMP9 (Table 2). MMP9 affects immunosuppressive features of the TME by inhibiting CD8+ T-cell infiltration and cytotoxicity, leading to resistance against anti-PD-1 therapy [125]. In CRC, KDM4D upregulates PD-L1 through JAK-STAT3 signaling, thus reducing CD8+ T-cell effectiveness and facilitating immune evasion (Figure 4). Mechanistically, KDM4D acts as a co-activator of SP1 to mediate IFNGR1 expression and, subsequently, enhance STAT3-IRF1 signaling and PD-L1 levels in a demethylase-dependent manner. Furthermore, pharmacological inhibition of KDM4D combined with PD-L1 antibodies impairs tumor growth and increases CD8+ T-cell infiltration and function [123,124]. Overall, KDM4-mediated influence on oncogenic signaling and immune evasion makes them critical targets for therapies to suppress tumor growth and enhance immunotherapeutic efficacy.

### 6.3. KDM7B/PHF8 Histone Demethylase

KDM7B, also known as histone demethylase PHD finger protein 8 (PHF8), operates by demethylating mono- and/or di-methylated lysines 9, 20, and 27 on histone H3 (H3K9me1/2, H3K20me1, and H3K27me2, according to the Uniprot database record Q9UPP1, accessed on 8 May 2024). Modulating histone methylation by KDM7B plays a critical role in neuronal differentiation [126]. Moreover, KDM7B is a vital promoter of metastasis in melanoma (Figure 4). Through its demethylase activity, KDM7B upregulates TGFß signaling and the expression of metastasis-related genes, thus promoting cell invasion and dissemination [127]. KDM7B overexpression is frequently observed across multiple cancer types and may play a crucial role in immune escape mechanisms (Figure 4, Table 2) [86]. As described above, KDM7B may stabilize SETDB1 independently of its demethylase activity. The abrogation of KDM7B results in the degradation of SETDB1, which in turn alleviates H3K9me3 repression on retroelements (REs). RE activation triggers viral mimicry response and enhances antitumor immunity [86].

**Table 2:** The effect of H3K9me3 demethylases on immune responses and tumor immunotherapy. A summary of observations linking H3K9me3 demethylases with various molecular mechanisms implicated in the immune response, and as such, affecting immunotherapy outcomes. Please see the text for details. GAFCP: gemcitabine (G), abraxane (A), CD40 agonistic antibody (F), CTLA4-blocking antibody (C), and PD–1–blocking antibody (P).

Histone Demethylase	Associated Genes or Pathways	Immune Response Effect of Active Demethylase	Effect on Tumor Immunotherapy	References
KDM3A	KLF5/SMAD4, EGFR	Lower population of T cells within the TME.	Resistance to combination GAFCP immunotherapy.	[117]
KDM3B	-	A positive correlation with resting mast cells, dendritic cells, and M2 macrophages. A negative correlation with regulatory T cells, follicular helper T cells, and M0 macrophages.	Not tested	[115]
KDM4A-D	-	Immune evasion via protection against replication stress and subsequent activation of the cGAS–STING pathway.	Drug JIB-04 (KDM4A-D inhibitor) improves the outcomes of anti-PD-1 therapy by triggering replication stress, which activates the cGAS-STING/IFN response, promoting CD8+ T-cell infiltration and immunogenic cell death.	[34,121]
KDM4C	ARID3B, PD-L1	Recruited by ARID3B, contributes to the upregulation of PD-L1, thus promoting immune evasion.	Not tested	[122]
KDM4D	Mutated CTNNB1, MMP9, SP1, PDL1	Together with CTNNB1^GOF^, activates MMP9 expression, which mediates an immunosuppressive TME by reducing CD8+ T-cell infiltration and cytotoxicity.	Mediates resistance to anti-PD-1 therapy.	[125]
KDM7B	SETDB1, TGFß signaling	Promotes SETDB1 stability, which leads to H3K9me3-mediated inhibition of retroelement (RE) activity and suppressed viral mimicry response.	Deletion of KDM7B can enhance the effectiveness of anti-PD-1 treatment via activation of the viral mimicry response.	[86]
LSD1	-	Low RE activity, suppressed viral mimicry response.	LSD1 inhibition in tumor cells improves the efficacy of anti-PD-1 therapy by RE activation and the subsequent viral mimicry response.	[128]

### 6.4. LSD1/KDM1A Histone Demethylase

Lysine-specific demethylase 1 (LSD1), also known as KDM1A, plays crucial roles in cancer initiation, progression, metastasis, and recurrence by removing lysine methylation marks from nucleosome histone tails [129,130]. LSD1 exhibits opposing epigenetic functions, as it removes methyl groups from activating H3K4me2/1 and repressive H3K9me2/1 [130]. This dual functionality underscores the pleiotropic influence of LSD1 across various cellular processes. LSD1 is overexpressed in many tumor types. It may modulate various signaling pathways in tumor and immune cells, supporting tumor epigenetic plasticity to evade therapeutic pressures and immune detection (Figure 4). Corresponding to this feature, growing evidence suggests that LSD1 inhibition may improve the outcome of immunotherapy, as recently reviewed in [129,130]. Notably, suppressing LSD1 in tumor cells enhances antigen processing and presentation, making them more detectable by the immune system [129]. The loss of LSD1 function may increase H3K4 dimethylation at RE loci and activate their expression, thus triggering the viral mimicry phenomenon (Figure 4, Table 2) [128]. In immune cells, LSD1 inhibition enhances T-cell activation, indicated by elevated CD69 expression on CD8+ T cells and increased secretion of cytokines, such as IL-2, IFN-γ, and TNF-α. Furthermore, LSD1 inhibition affects macrophages, NK cells, and cancer-associated fibroblasts, transforming “cold tumors” into “hot tumors” that are more susceptible to immune attack [129].

Despite promising findings on LSD1 inhibition in immunotherapy, several challenges remain. The exact molecular mechanisms by which LSD1 influences T-cell functions are not fully understood. Specifically, while many reports focus on the H3K4 demethylase activity of LSD1, the precise contribution of H3K9 and H3K4 signaling to the mode of action of LSD1 in the immune response needs further investigation. Additionally, the consistency of these effects across different solid tumors must be validated, and potential side effects, such as T cells attacking normal cells, require a thorough examination to ensure the safety of LSD1 inhibition [130].

## 7. H3K9me3 Readers

### 7.1. HP1/CBX

The mammalian HP1 family includes three members: HP1ß, HP1γ, and HP1α (also known as chromobox protein homolog, respectively: CBX1, CBX3, and CBX5). All of them are readers of H3K9me3 marks and effectors of heterochromatin formation. H3K9me3 attracts HP1 to particular locations in the genome, where it mediates the formation of heterochromatin, chromatin packing, DNA damage repair, and/or gene repression [131,132]. HP1 isoforms have been shown to be implicated in normal and tumor stem cell biology [133,134]. Although the expression level of HP1 isoforms varies across cancer types, it is often higher than in healthy tissues. In cancer, altered HP1 expression is positively associated with tumorigenic features, such as cancer proliferation, invasion, and metastasis [132,135,136]. Upregulation of HP1 proteins is frequently associated with poor survival rates; however, the overall outcome depends on the cancer type and HP1 isoform [137,138]. Moreover, emerging data pinpoint HP1 engagement in cancer immune status (Table 3).

With minor exceptions, the data published so far agree that HP1 expression positively correlates with CD4+ T-cell infiltration. However, the strength of the association between distinct HP1 isoforms and immune cell infiltration varies in different cancer types. In certain cases, the association is insignificant [139,140,141,142,143], while the only negative correlation was observed for HP1γ and the CD4+ T cell populations in esophageal cancer [143] and pancreatic cancer [141]. The most limited data on the effect of a specific HP1 isoform on cancer-immune interactions are available for HP1ß. Both HP1α and HP1ß positively correlate with the expression of PD-L1 immune checkpoints [132,144]. The observations reported by Kloetgen and colleagues indicated that HP1α suppresses the genes involved in inflammation, apoptosis, and death receptor signaling [145]. In the study conducted by Zhao and colleagues on nasopharyngeal carcinoma, targeting HP1ß inhibited STAT1 activation via IFNγ, which led to lowered PD-L1 expression and, as such, enhanced cancer cell killing by CAR T-cells [132].

More data are available for HP1γ. For instance, the data from Prof. To-Ha Thai’s team revealed that HP1γ insufficiency in CD8+ T cells augmented their effector capacity. This phenomenon was ascribed to reduced H3K9me3 deposition at regulatory regions of various immune-related genes, including *IL-21R*, *IFNγ*, *perforin*, and *granzyme B*. The epigenetic de-repression of these genes supported the expansion of CD8+ T cells, TME conversion to ”hot”, the immunogenic phenotype, and tumor growth inhibition in HP1γ-deficient mice bearing ovarian tumors or neuroblastoma cells [146,147]. In a study utilizing colon inflammation and colorectal cancer models, HP1γ was found to reside on *STAT1* and *CD274* promoters to mediate their transcriptional repression, which is alleviated upon IFNγ signaling [148].

By contrast, a positive correlation was observed between HP1γ expression and PD-1, PD-L1, and PD-L2 levels in gastric cancer. HP1γ expression was also related to TMB and neoantigen count. The study also revealed that high HP1γ expression was negatively correlated with the levels of tumor-infiltrating lymphocytes (TILs), including B cells, CD4+ and CD8+ T cells, neutrophils, macrophages, and DCs [131]. In clear cell renal carcinoma, HP1γ expression positively correlated with immune receptor activity, lymphocyte immunity, and several steps within the immunity cycle, including the release of cancer antigens, priming and activation, immune cell recruitment, cancer cell recognition, and eradication. In particular, a high HP1γ level was associated with the infiltration of macrophages, memory B cells, and plasmacytoid DCs. It was proposed that such a contribution of HP1γ to cancer immunity may be exerted via activation of PI3K/AKT signaling [135].

Moreover, by utilizing pan-cancer datasets, Xu and colleagues showed that the expression of HP1γ negatively correlated with infiltration of the majority of immune cell types. In addition, an inverse association was also observed between HP1γ and the expression of immune-related genes and the activity of immune pathways. Thus, the data indicate that high HP1γ expression is associated with an immunosuppressive TME [149]. The reported data focus on various HP1 isoforms and research models and show the differential influence of HP1 on immune signaling. However, most reports point toward a negative effect of HP1 on immune-related genes, which links HP1 with immunosuppression. Notably, some of the reports ascribed the immunoinhibitory effect of HP1 isoforms to the H3K9me3 heterochromatin mark.

### 7.2. KAP1/TIF1-β/TRIM28

Krüppel-associated box (KRAB)-associated protein 1 (KAP1), also known as transcriptional intermediary factor 1 β (TIF1-β) or tripartite motif containing 28 (TRIM28), is a member of the TRIM family genes, which are involved in several cellular processes. TRIM28 contains multiple domains and residues that are subject to post-translational modifications facilitating oligomerization, E3 ligation, and, importantly, interaction with other proteins. It is a central component of the repressive complex (combining KRAB-ZNF genes, SETDB1, and HP1) that confers H3K9me3 deposition and heterochromatization. It may interact with many other protein partners to exert its pleiotropic functions. It participates in various cellular processes, such as anti-(endo)virus response, DNA damage repair, immune signaling, stemness, differentiation, and carcinogenesis [150,151].

**Figure 5:**
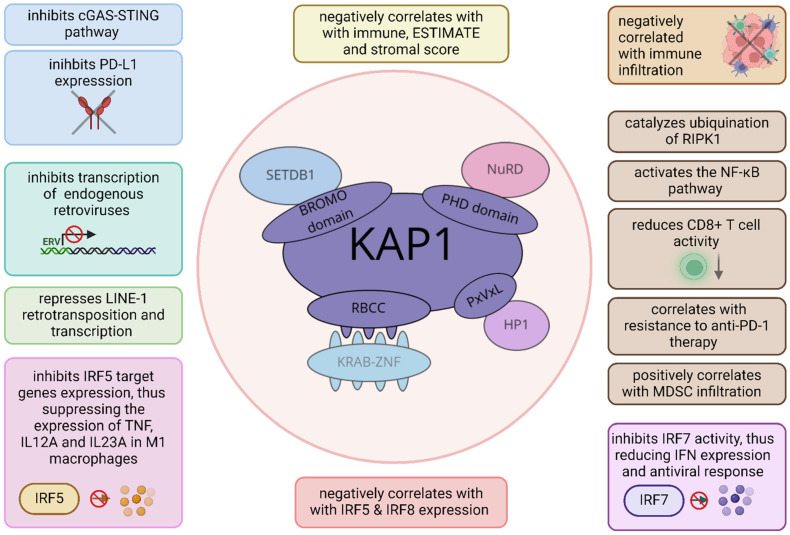
KAP1 affects the immune response with a wide range of molecular mechanisms. It inhibits the cGAS–STING pathway and PD-L1 expression and negatively correlates with immune cell infiltration. In addition, KAP1 ubiquitinates RIPK1 and activates the NF-κB pathway. It also reduces CD8+ T-cell activity, lowers sensitivity to PD-1 blockade, and increases MDSC infiltration. Moreover, KAP1 inhibits IRF factors, which leads to reduced cytokine release. It may also impair transcription of various transposable elements, e.g., LINE1 and endogenous retroviruses. Created in BioRender. Oleksiewicz, U. (2024) BioRender.com/i05b268, accessed on 12 October 2024.

Generally, the expression of KAP1 is elevated in various cancer types compared to healthy tissue [152,153,154], while cancer patients with higher KAP1 expression have a worse prognosis [153,154]. The role of KAP1 in cancer is complex but mostly oncogenic. It facilitates cancer progression, promotes DNA damage repair, and affects the interactions between cancer and the immune system (Figure 5, Table 3) [33,151,155].

As described above, the KAP1/SETDB1 complex plays a crucial role in suppressing antitumor immunity in ovarian cancer. Depleting this complex results in the activation of cGAS–STING and PD-L1 expression, potentiating ICB treatment efficacy [33]. Another complex, KAP1/ZNF689, binds to the LINE1 promoter to foster its H3K9me3-dependent transcriptional silencing. The loss of ZNF689 activated LINE1 retrotransposition, thus exacerbating intratumoral heterogeneity. These molecular events weakened antigen presentation, impeded T-cell infiltration, and decreased sensitivity to immunotherapy in triple-negative breast cancer [155]. In lung cancer, KAP1’s influence on cancer immunity is associated with its E3 ligase activity. KAP1 catalyzes the ubiquitination of RIPK1 (receptor-interacting protein kinase 1), activating the NF-κB pathway and promoting the chemokine-driven recruitment of immunosuppressive MDSCs. This process also attenuates CD8+ T-cell activity and enhances anti-PD-1 resistance [152]. Collectively, KAP1 knockout may enhance the efficacy of anti-PD-1 therapy. In addition, KAP1 was shown to be a promising target in radiotherapy, where its deletion enhanced radiation-induced REs activation, subsequent cytosolic DNA release, and stimulation of the cGAS/STING pathway. These processes evoke immune mechanisms that augment radiation-induced cell death [156].

It is likely that KAP1’s contribution to the interactions between cancer and the immune system is much wider and, possibly, context-dependent, as it has been shown to influence the immune response in multiple ways. A study by Eames and colleagues revealed that in M1 macrophages, KAP1 mediates H3K9me3 deposition on IRF5 target genes, thus suppressing the expression of pro-inflammatory cytokines (e.g., TNF) [39]. Through its sumoylation activity, KAP1 also hinders the transcriptional activity of IRF7 to attenuate IFN expression and antiviral responses [157]. KAP1 was also shown to mediate the sumoylation of other IRF family members (IRF5 and IRF8), likely leading to an immunosuppressive TME in lung adenocarcinoma [158]. Indeed, KAP1 expression was negatively correlated with immune, ESTIMATE, and stromal scores in several cancer types [154,158]. In conclusion, KAP1 is an important regulator of cancer formation and the immune response, whereas its overexpression is associated with a bad prognosis. Targeting KAP1 is a promising strategy for enhancing the efficacy of cancer treatment.

### 7.3. CHD3/4

CHD, a chromodomain helicase DNA-binding family, is a subfamily of ATP-dependent chromatin-remodeling proteins. This family consists of nine members divided into three subclasses. In contrast to other subclasses, subclass 2 belongs to H3K9me3 readers containing plant homeodomains (PHDs). Subclass 2 consists of CHD3, CHD4, and CHD5 [51], which may be recruited within the NuRD complex [54]. CHD3 and CHD4, also known as Mi-2α and Mi-2β, play roles in transcriptional repression and DNA damage signaling [55,159]. Many cancer types present increased CHD4 levels but variable levels of CHD3. Overexpression of both proteins is associated with poor prognosis [55]. Most of the data published to date related to the involvement of these proteins in cancer immunology focus on CHD4.

It is believed that CHD4, together with its protein partners (e.g., from the NuRD complex), has an oncogenic role in cancer, as it promotes cell growth, migration and invasion, metastasis, and DNA damage repair via silencing various tumor suppressor genes [160,161]. Knockdown of CHD4 was shown to augment radiosensitivity by aggravating DNA damage [162] and promote chemotherapy and drug sensitivity [163,164,165].

Based on bioinformatics analyses of TCGA datasets for hepatocellular cancer, CHD4 expression was found to negatively correlate with the complement and coagulation pathways. A similar inverse association was identified between CHD4 levels and the infiltration of CD8 + T cells and DCs, suggesting an adverse influence of CHD4 on tumor immunosurveillance (Table 3) [160]. Consistently with these findings, CHD4 was found to act as an inhibitor of CD8+ T cells, IFNγ-stimulated genes, and IFNα signaling. CHD4 insufficiency reduced immune suppression, as could be observed by elevated CD4+ and CD8+ T cell populations within the tumor, and enhanced the effectiveness of antitumor T-cell responses in melanoma-bearing mice treated with an anti-PD-1 antibody. Of note, the inhibitory effect of CHD4 on immune signaling was mediated through K510 methylation of EZH2, which increased its H3K27 methylation activity and led to the epigenetic repression of IFNγ target genes [166]. In colorectal cancer, CHD4 was found to recruit G9a H3K9 methyltransferase to suppress galectin-7 expression. These processes repress CD8+ T-cell activity, resulting in poorer anti-PD1 treatment outcomes [167].

### 7.4. CDYL

CDYL, an epigenetic factor chromodomain Y-like, is a member of chromatin Y-related proteins. It contains an N-terminal chromodomain (recognizing H3K9me3 and H3K27me2/3), a region for EZH2 interaction, and a C-terminal acetyl-coenzyme A-binding domain (based on Uniprot record Q9Y232, accessed on 8 May 2024). CDYL functions as a transcriptional co-repressor. It is an important factor in neuronal development and maintenance of neuroplasticity [168,169].

CDYL expression was shown to be elevated in glioma [168] and in chemoresistant small-cell lung carcinoma, where its overexpression was associated with an advanced clinical stage and poor prognosis [170]. The insights into CDYL function in cancer immunity remain limited. However, in a study by Kong and colleagues [168], CDYL revealed an association with immunosuppression within glioma cells (Table 3). The data indicated that CDYL depletion decreased glioma development and promoted immune pathways and expression of chemokine ligands (e.g., chemokine [C-C motif] ligand 2 [CCL2]). More specifically, CDYL knockdown evoked the influx of CCL2-recruited monocytes/macrophages and M1-like TAMs (tumor-associated macrophages/microglia) with antitumor activities. Simultaneously, CDYL knockdown restricted the presence of tumor-promoting M2-like TAMs. The authors proposed that CDYL may serve as a potential candidate therapeutic target in glioma [168]. However, future studies are required to elucidate the molecular mechanisms behind CDYL functioning.

### 7.5. UHRF1

UHRF1 (ubiquitin-like, containing PHD and RING finger domains 1), a 793 amino acid protein, includes five essential domains: ubiquitin-like (UBL), tandem tudor (TTD), PHD, SET and RING associated (SRA), and really interesting new gene (RING) [171]. The UBL domain stabilizes UHRF1 and recruits DNMT1, while the other domains facilitate DNA methylation, chromatin recognition, and ubiquitylation [172,173]. UHRF1 maintains epigenetic stability by coordinating DNA and histone methylation [174]. In addition, it binds histone modifications (specifically, H3K9me3) and recruits other proteins involved in transcriptional repression, such as HDAC1, DNMT1, G9a, and SUV39H1 [175,176,177].

The transcriptional co-repressor function of UHRF1 was illustrated in experiments conducted on glioma and mesothelioma cells, in which UHRF1 contributed to the epigenetic silencing of NY-ESO1, a cancer-testis antigen regarded as a target for cancer immunotherapy. The silencing required sequential epigenetic regulation by multiprotein complexes: HDAC1-mSIN3a-NCOR, DNMT3B-HDAC1-EGR1, and DNMT1-PCNA-UHRF1-G9a. Such cooperation between epigenetic modifiers mediated a profound epigenetic rearrangement of the *NY-ESO1* locus that involved histone deacetylation, histone methylation, and DNA methylation [178]. The co-repressor function of UHRF1 triggering the epigenetic silencing of genes involved in the immune response was also evidenced in human papillomavirus-negative head and neck squamous cell carcinomas (Table 3). In the study by Nigam and colleagues, lysine methyltransferase SET and MYND domain containing 3 (SMYD3) was shown to bind the *UHRF1* promoter to activate its transcription. Upregulated UHRF1 interacted with H3K9me3-enriched regulatory regions of immune-related genes (e.g., type I IFN response genes) and recruited DNMT1 to inhibit their expression. Depletion of Smyd3 potentiated CD8+ T-cell infiltration and anti-PD-1 therapy in syngeneic mouse mode, indicating Smyd3/Uhrf1’s contribution to immunosuppression [177]. Another study also confirmed that UHRF1 may promote the immunosuppressive features of tumor cells through the maintenance of functional Tregs. UHRF1 silencing lowered forkhead box P3 (FOXP3) expression and triggered cell death in Tregs. However, the exact mode of UHRF1 action remains to be further explored [179].

**Table 3:** The influence on cancer immune phenotype conveyed by H3K9me3 readers in various cancer types. The research models used in the specific research is indicated in the table.

	Mechanisms and Phenotypes	Cancer Types	Research Models	References
HP1	HP1α positively correlated with CD4+ T cells.	ovarian cancer	TIMER2.0 database	[139]
	All HP1 isoforms positively correlated with CD4+ T cells.	head and neck squamous cell carcinoma	TIMER2.0 database	[140]
	HP1γ negatively correlated with CD4+ T cells.	pancreatic carcinoma	TIMER2.0 database	[141]
	HP1β and HP1α positively correlated with CD4+ T cells.	colon cancers	TIMER2.0 database	[142]
	HP1β and HP1α positively correlated with CD4+ T cells. HP1γ negatively correlated with CD4+ T cells.	esophageal cancer	TIMER2.0 database	[143]
	YBX1 knockdown and subsequent HP1α mRNA instability led to the upregulation of genes involved in the inflammatory response, apoptosis, and death receptor signaling.	medulloblastoma	medulloblastoma cell lines: DAOY, Med8A, and UW228-3	[145]
	HP1γ insufficiency in CD8+ T cells led to higher transcriptional activity of immune-related genes, increased effector capacity, CD8+ T-cell expansion, remodeled the TME, and tumor rejection.	ovarian cancer, neuroblastoma, melanoma	HP1γ-deficient mice, Rag2^−/−^cγ^−/−^ mice ovarian cancer cell lines: ID8 and MOSE-L_TICv_, neuroblastoma cell line: NB9464, melanoma cell line: B16-F10	[146,147]
	HP1β inhibition suppressed IFNγ-induced STAT1 activation, which reduced PD-L1 expression and enhanced cancer cell killing by CAR T-cells.	nasopharyngeal carcinoma (NPC)	GSE102349 dataset NPC cells lines: SUNE1, HONE1, HK1 NPC tissue samples from patients	[132]
	HP1γ depletion increased the expression of STAT1 and PD-L1 upon IFNγ stimulation.	colon inflammation and colorectal cancer	colorectal cancer cell lines: HT29, SW480, and HCT116 mice HP1γ KO colon epithelium	[148]
	HP1γ negatively correlated with the levels of B cells, CD8+ and CD4+ T cells, macrophages, neutrophils, and dendritic cells. HP1γ positively correlated with tumor mutation burden, neoantigen count, and PD-1, PD-L1, and PD-L2 levels.	gastric cancer	TIMER2.0 database, TCGA database	[131]
	HP1γ positively correlated with immune receptor activity, lymphocyte immunity, cancer antigen release, priming and activation of immune cells, recognition and killing of cancer cells by T cells, and the infiltration of macrophage, memory B cells, and plasmacytoid dendric cells.	clear cell renal carcinoma	TCGA database and Kyoto Encyclopedia of Genes and Genomes (KEGG)	[135]
	HP1γ positively correlated with the levels of Th/Th2 cells, T central memory cells, and Tγδ cells and negatively correlated with 20 other immune-related cells. HP1γ negatively correlated with the expression of most immune-related genes and TME score.	multiple tumor types	Pan-cancer TCGA datasets, Human Protein Atlas (HPA), cBioPortal and Genotype Tissue-Expression (GTEx)	[149]
KAP1	KAP1/SETDB1 inhibition activated the cGAS–STING pathway, thus enhancing PD-L1 expression, immune cell infiltration, and the efficacy of anti-PD-L1 treatment.	ovarian cancer, melanoma	ovarian cancer cell lines: ID8 and UPK10 (mouse), OVCAR3 (human) orthotopic mouse model C57BL/6 Cancer Cell Line Encyclopedia (CCLE), TCGA, Public Melanoma Proteomics Dataset	[33]
	ZNF689 repressed LINE-1 retrotransposition via KAP1 complex-mediated transcriptional silencing. ZNF689 deficiency increased LINE-1 retrotransposition, which augmented genomic instability and intratumor heterogeneity. This in turn impaired antigen presentation, decreased T-cell infiltration, and sensitivity to immunotherapy.	triple-negative breast cancer	triple-negative breast cancer cell lines: LM2, Hs578T, MDA-MB-231 (human), 4T1, AT3 (mice) tumors from four triple-negative breast cancer trials treated with anti-PD-1 therapy (n = 109).	[155]
	KAP1 depletion inhibited RIPK1 ubiquitination and reduced NF-κB-mediated CXCL1 release, thus limiting MDSC infiltration in syngeneic murine tumors. KAP1 overexpression caused bigger tumor sizes, higher MDSC infiltration, and decreased CD8+ T-cell presence in the TME. It conferred resistance to anti-PD-1 therapy.	non-small cell lung cancer	lung cancer cell lines: CMT-167 and H1299. KrasLSL−G12D/+; Tp53fl/fl C57BL/6J mice models	[152]
	KAP1 negatively correlated with immune cell infiltration.	pan-cancer analysis	Tumor Immune Estimation Resource (TISIDB) and TIMER	[158]
	KAP1 negatively correlated with IRF5 and IRF8 expression and with stromal and immune scores. KAP1 knockdown led to higher levels of IRF5 and IRF8.	lung adenocarcinoma	TCGA, GSE43580, GSE7670 Lung adenocarcinoma cell lines PC9 and H1299	
CHD3/4	CHD4 negatively correlated with complement and coagulation pathways and infiltration of CD8+ T cells and dendric cells.	hepatocellular carcinoma	TCGA datasets	[160]
CDYL	CDYL deficiency augmented the antitumor response and hindered tumor growth in vivo. It resulted in increased expression of chemokine ligands (CXCL12, CCL2), which contributed to monocyte and macrophage recruitment. CDYL knockdown elevated the influx of M1-like tumor-associated macrophages/microglia (TAMs), and decreased the population of M2-like TAMs.	glioma	human glioblastoma cell line U87MG nu/nu nude mice, CB-17 SCID mice, NOD SCID mice THP-1 monocytes	[168]
UHRF1	UHRF1 interacts with H3K9me3 at the promoters of immune-related genes (e.g., CXCL10, CXCL9) and forms a complex with DNMT1 that catalyzes DNA methylation to mediate epigenetic repression.	HPV negative head and neck squamous cell carcinoma	Tongue squamous cell carcinoma cell line HN-6	[177]
	UHRF1 depletion in Tregs decreased FOXP3 expression, thus inducing Treg apoptosis and limiting their suppressive effect on the proliferation of conventional T cells.	regulatory T cells (Tregs)	Tregs isolated from the blood of healthy donors	[179]

## 8. Conclusions

Albeit very promising, cancer immunotherapy still faces numerous challenges. Accelerating interest and research on H3K9me3 modification continues to unveil its complex role in the regulation of cancer cells, influencing the tumor microenvironment and orchestrating the immune response. A growing body of evidence indicates that most H3K9me3 modifiers (both methyltransferases and demethylases) and readers impede immunotherapy, and different mechanisms contribute to this phenomenon. Many methyltransferases guard RE repression, while their deficiency leads to the loss of H3K9me3, RE upregulation, and viral mimicry mechanisms that turn on the cGAS–STING pathway and IFN signaling. Interestingly, KDM4A knockout also triggers cGAS–STING and IFN signaling, however as a result of increased H3K9me3/HP1 puncta on chromatin that stall DNA replication, thus unleashing replication stress. Moreover, replication stress mediating cGAS–STING signaling can also be evoked by aberrant G9a/UHRF1 functioning. The opposite effects of H3K9me3 levels eliciting similar outcomes highlight the importance of the intricate balance of this epigenetic mark in immune-related processes.

While cGAS–STING upregulation is one of the immune system-activating mechanisms, the other mechanism involves deregulating specific immune-related genes targeted by H3K9me3 modifiers. Although, in some cases, these downstream genes were shown to be regulated in an H3K9me3-dependent manner, in other reports, the regulation mechanism was either insufficiently explored or the mode of action was not associated with H3K9me3. Conversely, in some cases, the influence on the immune system was ascribed to the methylation of other histone and non-histone lysines due to the broader specificity of H3K9 modifiers (e.g., H3K4me3, as in the case of LSD1). In addition, H3K9me3 readers are large proteins, frequently containing a few binding and catalytic domains. Thus, their spectrum of actions is much wider than the H3K9me3 interpretation. Moreover, the activities of H3K9me3 modifiers and readers depend on the molecular context, as shown in the case of KDM4D in ß-catenin-mutated hepatocellular carcinoma.

Collectively, in most of the cases, the expression of H3K9 modifiers and effectors is inversely correlated with canonical immune signatures (e.g., IFN, TNFα/NF-κB signaling, inflammatory response, JAK/STAT pathway) and cytolytic features, which characterize immune-excluded tumors (i.e., cold tumors). These tumors are notoriously resistant to immunotherapies and thus present a major challenge in modern oncology. Overall, the reviewed data indicate that H3K9me3-related epigenetic factors have great potential to overcome the hurdles posed by immune escape and hold promise for successful anticancer immunotherapies.

## Data Availability

Not applicable.
